# Mass spectrometry imaging of Alzheimer's disease vulnerable and resilient neocortical areas in postmortem formalin‐fixed paraffin‐embedded and fresh frozen human brains

**DOI:** 10.1002/alz70855_105665

**Published:** 2025-12-24

**Authors:** Tassia Venga Mendes, Patrick R Hof, Daniel Meyer, Merina Varghese, Livia Schiavinato Eberlin

**Affiliations:** ^1^ Baylor College of Medicine, Houston, TX, USA; ^2^ Icahn School of Medicine at Mount Sinai, New York, NY, USA; ^3^ GE HealthCare, Niskayuna, NY, USA; ^4^ University of Rhode Island, Kingston, RI, USA

## Abstract

**Background:**

Alzheimer's disease (AD) is a progressive neurodegenerative disorder characterized by amyloid‐beta, tau pathology, neurodegeneration, and metabolic changes. Vulnerable neocortical areas, such as dorsolateral prefrontal cortex (DLPFC), are significantly affected early in AD, while resilient areas, like primary visual cortex (V1), remain relatively unaffected till later in the disease. Spatial characterization of the molecular changes in different neocortical areas is key to understanding factors involved in disease progression. Here, we employed Desorption Electrospray Ionization Mass Spectrometry Imaging (DESI‐MSI) to investigate metabolite and lipids signatures in tissue microarrays of formalin‐fixed paraffin embedded (TMA FFPE) and fresh frozen brain tissues. The objective is to optimize experimental methods and spatially characterize AD‐related molecular changes in DLPFC and V1 at different disease stages.

**Method:**

Postmortem brain tissues from DLPFC (healthy, frozen) and V1 (AD, TMA FFPE) were analyzed. TMA FFPE tissue underwent xylene‐based deparaffinization and ammonium treatment before DESI‐MSI using a Xevo G2‐XS QTOF at 100 µm spatial resolution in positive and negative ion modes. Optimization experiments focused on comparing molecular recovery between TMA FFPE and frozen samples.

**Result:**

We detected 97 metabolites in frozen DLPFC and 29 in TMA FFPE V1 by DESI‐MSI on negative mode. Pyruvic acid, lactic acid, and L‐serine were detected in both formats, while gamma‐aminobutyric acid, creatine, and uric acid were only detected in frozen tissues. Serine, ribose‐5‐phosphate, and hexacosapentaenoic acid were only detected in TMA FFPE samples. Regarding fatty acids, arachidonic acid, eicosapentaenoic acid, and docosahexaenoic acid were detected in both. Preliminary analysis of TMA FFPE DLPFC is ongoing in negative mode, as well as in positive mode for frozen and both TMA FFPE DLPFC and V1 neocortical areas.

**Conclusion:**

This study demonstrates the viability and limitations of TMA FFPE compared with frozen tissues to map brain molecules by MSI. Despite frozen tissues yielding higher metabolite recovery, FFPE tissues are widely available in brain banks and more easily integrated with histology and immunofluorescence techniques. Thus, this spatial MSI is a valuable tool to evaluate molecular changes more broadly across brain regions in AD, offering insights into disease progression and correlated biomarkers.

